# Rapid Detection of *Neisseria gonorrhoeae* Genomic DNA Using Gold Nanoprobes Which Target the Gonococcal DNA Uptake Sequence

**DOI:** 10.3389/fcimb.2022.920447

**Published:** 2022-07-08

**Authors:** Ella Carter, Sean A. Davis, Darryl J. Hill

**Affiliations:** ^1^ School of Chemistry, University of Bristol, Bristol, United Kingdom; ^2^ Bristol Centre for Functional Nanomaterials, University of Bristol, Bristol, United Kingdom; ^3^ School of Cellular and Molecular Medicine, University of Bristol, Bristol, United Kingdom

**Keywords:** *Neisseria gonorrhoeae*, diagnosis, gold nanoparticle, point of care (POC) diagnosis, DNA uptake sequence

## Abstract

The rapid spread of antimicrobial resistant *Neisseria gonorrhoeae* continues to pose a serious threat to global health. To successfully treat and control gonococcal infections, rapid diagnosis is critical. Currently, nucleic acid amplification tests are the recommended diagnostic, however, these are both technically demanding and time consuming, making them unsuitable for resource-poor clinics. Consequently, there is a substantial need for an affordable, point-of-care diagnostic to use in these settings. In this study, DNA-functionalised gold nanoparticles (gold nanoprobes), with the ability to specifically detect the DNA Uptake Sequence (DUS) of *Neisseria gonorrhoeae*, were prepared. Using complementary annealing, the gold nanoprobes were shown to hybridise to genomic gonococcal DNA, causing a significant shift in their salt stability. By exploiting the shift in nanoprobe stability under the presence of target DNA, a solution-based colorimetric diagnostic for gonococcal DNA was prepared. Detection of purified genomic DNA was achieved in under 30 minutes, with a detection limit of 15.0 ng. Significantly, testing with DNA extracted from an off-target control organism suggested specificity for *Neisseria*. These results highlight the potential of DUS-specific gold nanoprobes in the rapid point-of-care diagnosis of gonococcal infections.

## Introduction

The sexually transmitted infection gonorrhoea presents a major global health burden, with approximately 100 million new cases every year (Blomquist et al., 2014). In addition to high infection rates, the causative bacterial species, *Neisseria gonorrhoeae* (*Ng*, gonococci) has shown an extraordinary ability to develop antimicrobial resistance, with resistance to almost all front-line antibiotics including sulfonamides, penicillins and fluoroquinolones (Costa-Lourenço et al., 2017; The World Health Organisation, 2020). Alarmingly, in recent years there has been a growing number of so-called “super-resistant” cases, in which the recommended therapy of extended-spectrum cephalosporins (ESCs) combined with azithromycin has failed (Eyre et al., 2018; The World Health Organisation, 2018; World Health Organisation, 2019a). Growing concerns over the future treatment and control of resistant gonococcal infections have prompted the World Health Organisation to classify *Ng* as a “Priority 2” pathogen (The World Health Organisation, 2016).

To ensure timely treatment of infections, rapid diagnosis is critical. Since the 1990’s, the gold standard for diagnosis of gonorrhoea is the use of nucleic acid amplification tests (NAATs), which offer good sensitivity and specificity (Whiley et al., 2006). However, these can be unsuitable in some resource poor or remote clinics due to the need for trained professionals and specialised laboratory equipment (Ng and Martin, 2005; Whiley et al., 2006). In addition, results typically take upwards of a week to be returned to the patient, which is particularly problematic in low to middle income countries (LMICs) where patient return rates are low (Vickerman et al., 2003). Moreover, many clinics in resource-poor settings rely on gram-strain based identification which provides poorer sensitivity and specificity than NAAT’s, often leading to a missed or mis-diagnosis (Bignell et al, 2006; Venkatesan and Griffin, 2009; Fredrick, 2012; Meyer and Buder, 2020). Such methods require overnight cell culturing and so results still take upwards of a day to be returned to the patient. Since STI prevalence is greatest in LMICs where access to suitable diagnostics is limited, there is a considerate need for a sensitive, affordable and rapid point-of-care diagnostic for gonococcal infections, that can be applied in these settings (Rowley et al., 2019; The World Health Organisation, 2019b).

Metallic nanoparticles (NPs; metal-based particles with dimensions less than 100 nm), have been extensively explored as markers for a range of point-of-care diagnostics. Most notably, gold nanoparticles (AuNPs) have been widely employed in pregnancy tests and more recently, COVID-19 lateral flow diagnostics (O’Farrell, 2008; Joysbio, 2020). AuNPs are a popular choice for diagnostic applications because of their facile synthesis and bioconjugation, high stability and unique optical properties (Zhou et al., 2015). In particular, AuNPs exhibit a characteristic surface plasmon resonance (SPR) which arises from collective oscillations of electrons in the conduction band of atomic gold (Vines et al., 2019). As a result, incident light which matches the frequency of this oscillation is strongly absorbed by the AuNPs. For spherical AuNPs below 100 nm, this absorption occurs in the visible region, and so the AuNPs exhibit a characteristic colour (Huang and El-Sayed, 2010). Aggregation of AuNP suspensions results in dipole-coupling between adjacent particles, causing a red-shift in the SPR to longer wavelengths (Huang and El-Sayed, 2010). Hence, AuNP aggregation results in a distinct colour change and has been widely exploited for the colorimetric detection of whole pathogens, proteins, DNA and RNA (Rajendran and Ellington, 2008; Zhang et al., 2012; Parolo et al., 2013; Liu et al., 2015; Lavu et al., 2016).

Herein, DNA-functionalised gold nanoparticles (gold nanoprobes) will be used to prepare a rapid, colorimetric diagnostic for *Neisseria gonorrhoeae*. More specifically, the nanoprobes are designed to detect the gonococcal DNA Uptake Sequence (DUS), a 12 base repeating motif (5’- ATGCCGTCTGAA-3’) present up to 2000 times in a single genome (Goodman and Scocca, 1988; Ambur et al., 2007; Duffin and Seifert, 2010; Frye et al., 2013). The DUS is known to facilitate highly-selective intraspecies transformation, resulting from specific recognition and binding of DUS-containing DNA to ComP within gonococcal type IV pili (Duffin and Seifert, 2012; Cehovin et al., 2013; Frye et al., 2013). This strong preference for intraspecies DNA is a unique characteristic only observed in the *Neisseriaceae* and *Pasteurellaceae* families (Graves et al, 1982). By targeting the gonococcal 12-base DUS, it was proposed high bacterial specificity would be achieved. In addition, it was hypothesised that the high DUS frequency would enhance the diagnostic sensitivity compared to previously reported approaches using gold nanoprobes (Liandris et al., 2009; Andreadou et al., 2014; Tunakhun et al., 2019).

## Materials & Methods

### Preparation and Characterisation of Gold Nanoparticles

Citrate-capped gold nanoparticles (AuNPs) were prepared using the Turkevich-Frens method (Kimling et al., 2006). Gold (III) chloride trihydrate (10 mg, 0.025 mmol) was dissolved in 95 mL Milli-Q water and heated to a rolling boil with continuous stirring. 5 mL of 1% w/v sodium citrate dihydrate solution was added, and the mixture heated for 15 minutes, resulting is a colour change from pale yellow to a dark red solution. The AuNP solution was stored in the dark at 4°C for several months. UV-visible spectroscopy (UV-vis) was used to confirm successful preparation and determine the AuNP molar extinction coefficient and solution concentration. The completed reaction mixture was deposited onto a 3.05 mm copper 400 mesh glow-discharged carbon-coated grid and imaged by a JEOL 1400 transmission electron microscope (TEM). Measurements were taken using ImageJ software to determine the average particle diameter from the collected TEM images. Dynamic light scattering (DLS) was used to measure the average hydrodynamic diameter and zeta potential.

### Preparation and Characterisation of DNA-Functionalised Gold Nanoprobes

A disulfide protected 5’-thiolated oligonucleotide (5’-[ThiC_6_]aaaaaaaaaatgacc**atgccgtctgaa**caaac-3’, DUS motif shown as bold text) was purchased from Sigma Aldrich. Prior to use, the oligonucleotide was deprotected by reduction with tris(2-carboxyethyl)phosphine (TCEP). For this, 30 µM oligonucleotide was added to 3 mM TCEP in 0.02 M HEPES buffer, pH 7.0. The oligonucleotide was reduced for 1 hour at room temperature, before purification using a Microspin G-25 column which has been prewashed with 0.01 M phosphate buffer, pH 7.5. The deprotected oligonucleotides were then conjugated to the bare AuNPs using a slow salt-aging process adapted from the method outlined by Mirkin (Hurst et al., 2006). Freshly cleaved oligonucleotide (4 nmol) was added to 1 mL of freshly synthesised AuNP suspension (∼ 1 nM) and the solution brought to 9 mM phosphate buffer, pH 7.5 and 0.1% SDS and shaken for 30 minutes. Over 48 hours, 6 incremental additions of 2 M NaCl were used to slowly bring the reaction to 0.3 M NaCl. The solution was then shaken for a further 24 hours before centrifuging at 12,000 *g* for 25 minutes to pellet the gold nanoprobes. The nanoprobes were washed 3 times with 0.01 M phosphate buffer, 0.1 M NaCl, before redispersion in a storage buffer (1 mM phosphate buffer, 0.1 M NaCl and 0.1% SDS) at ∼ 10 nM. The suspension was stored at 4°C in the dark until further use.

The absorbance spectrum of the DNA-functionalised nanoprobe suspension was measured by UV-visible spectroscopy on a PerkinElmer Lambda 750. The average hydrodynamic particle diameter and zeta potential were measured by Dynamic Light Scattering (DLS) using a Malvern Zetersizer Nano. The nanoprobes were imaged using TEM. For this, dilute aqueous suspensions of the nanoprobes were deposited onto glow-discharged, carbon-coated 3.05 mm 400 mesh TEM grids and the excess liquid wicked away using filter paper. The TEM grids were then stained with 1% uranyl acetate for 30 seconds before washing three times with deionised water to create a positive stain. The grids were air dried overnight and imaged on a JEOL 1400 TEM.

To quantify the number of DNA strands attached to each particle, mercaptoethanol was used to cleave the gold-thiol bonds, thus detaching the DNA from the AuNP surface (Cordray et al., 2012). The solution was centrifuged at 14000 RPM and the supernatant combined with an Invitrogen™ Quant-iT™ OliGreen™ fluorescent reagent, designed specifically to bind and detect single-stranded oligonucleotides. Fluorescence emission was measured and compared to a calibration plot for different oligonucleotide concentrations, to determine the concentration of detached oligonucleotides.

### Salt-Induced Aggregation for the Detection of Complementary Oligonucleotides

Gold nanoprobe suspension (3 µL) was added to an equal volume of a reverse complement DUS oligonucleotide (5’- gtggt**ttcagacggcat**ttgtg-3’, motif shown as bold text, 5 random flanking bases included either side of the complement sequence) to give a final oligonucleotide concentration of 50 µM. A non-complementary oligonucleotide (5’-gtgattactgtccggaactgtg-3’) was used as a control. The suspensions were heated to 95°C for 5 minutes, cooled to room temperature for 15 minutes before adding MgSO_4_ to a final concentration between 0-100 mM. Each suspension was photographed, and particle aggregation observed visually. The UV-visible absorbance spectrum of each solution was collected between 400-700 nm using a PerkinElmer Lambda 750 UV-vis spectrophotometer. Each experiment was repeated 3 times.

### Testing the Sequence Specificity of Aggregation Tests

Gold nanoprobe suspension (3 µL) was added to an equal volume of partial matching complementary oligonucleotides to give final oligonucleotide concentrations of 50 µM. The mixtures were heated to 95°C for 5 minutes and after cooling at room temperature for 15 minutes, MgSO_4_ was added to give a final concentration of 40 mM. Particle aggregation was observed visually and by measuring the UV-vis absorbance of each solution between 400-700 nm. Each experiment was repeated 3 times.

### Salt-Induced Aggregation for the Detection of Gonococcal Genomic DNA

Genomic DNA (gDNA) was extracted and purified from wild-type MS11 *N. gonorrhoeae* and Stellar *Escherichia coli (E. coli)* as a control, using a Qiagen DNeasy Blood and Tissue Kit. 3 µL of gold nanoprobe was added to an equal volume of *N. gonorrhoeae* or *E. coli* DNA to give a final concentration between 1-30 ng µL^-1^. The mixture was heated to 95°C for 5 minutes and after cooling at room temperature for 15 minutes, MgSO_4_ was added to give a final concentration of 40 mM. Particle aggregation was observed visually and by measuring the UV-vis absorbance of each solution between 400-700 nm. Each experiment was repeated 3 times.

## Results

### Preparation and Characterisation of Gold Nanoprobes

**Figure 1:**
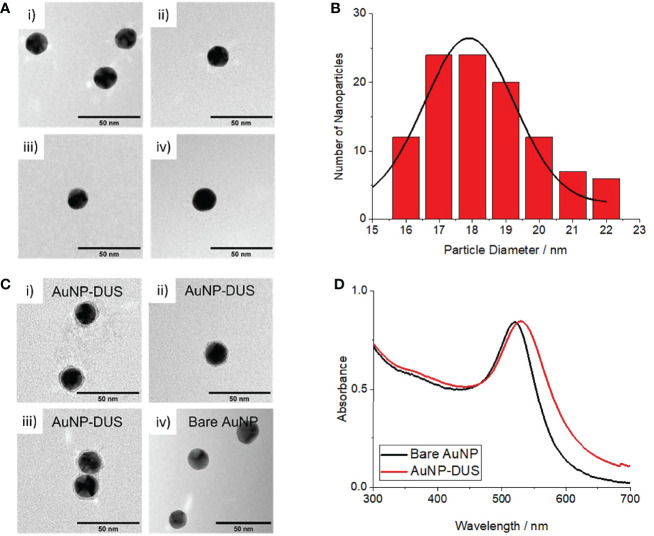
Characterisation of bare AuNPs and DNA-functionalised AuNPs (nanoprobes) **(A)** TEM images of bare AuNPs, i-iv) individual images taken from random position on the TEM grid **(B)** Histogram of measured particle diameters for bare AuNPs **(C)** i-iii) TEM images of AuNP-DUS with uranyl acetate staining showing DNA conjugated to the particles surface, iv) TEM image of bare AuNP with same uranyl acetate staining showing no peripheral staining, **(D)** UV-visible absorbance spectrum of bare AuNPs (black) and nanoprobes (red) showing a 9nm shift in surface plasmon resonance (SPR) upon DNA-functionalisation.

Spherical AuNPs with an average diameter of 18.3 ± 1.7 nm were successfully produced as observed by TEM (**Figures 1A, B**). Dynamic light scattering measurements revealed an average hydrodynamic diameter of 28.9 ± 0.4 nm and a large negative zeta potential (-29.0 ± 4.9), accounted for by the electrostatically absorbed citrate groups. The AuNPs exhibited a maximum absorbance at 520 nm (see black curve in **Figure 1D**) consistent with their predicted surface plasmon resonance (Link and El-Sayed, 1999).

Following surface-functionalisation of the AuNPs with a thiolated DUS-containing oligonucleotide, a 29 nm average increase in hydrodynamic diameter was observed, consistent with approximately two lengths of the solvated oligonucleotides. Conjugation of DNA around the particle surface was confirmed by TEM imaging, where uranyl acetate-stained DNA was observed around the periphery of the nanoprobes but not for the bare AuNPs (see **Figure 1C**). A small 9 nm increase in surface plasmon resonance was observed for the nanoprobes (see red curve in **Figure 1D**), likely due a combination of the influence of DNA on the local dielectric constant and refractive index at the nanoparticle surface (Thompson et al., 2008; Huang et al., 2019). This confirmed that the particles remained stable in solution following oligonucleotiode functionalisation. Cleavage of the gold-thiol bonds and subsequent quantification of the released DNA using a Quant-it OliGreen fluorescent reagent, revealed that the DUS functionalised nanoprobes contained approximately 145 oligonucleotides per particle.

### Salt-Induced Aggregation for the Detection of Complementary Oligonucleotides

**Figure 2:**
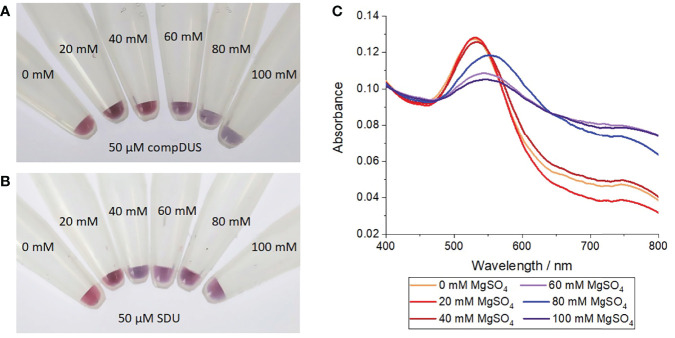
Salt-induced aggregation test of gold nanoprobes treated with complementary or control oligonucleotides. **(A)** Photograph of nanoprobes after incubation with 50 µM of a reverse complement DUS (compDUS), heating to 95°C, before cooling to room temperature and treating with different concentrations of MgSO4, **(B)** Photograph of nanoprobes after treatment as before but with 50 µM of random scramble oligonucleotide (SDU) in place of compDUS, **(C)** UV-vis absorption spectra of nanoprobe samples treated with compDUS at increasing concentrations of MgSO_4_. Each experiment was repeated 3 times, with photographs shown from 1 repeat.

Complementary annealing to a reverse complement DUS (compDUS) oligonucleotide conferred additional stability to the DUS-functionalised nanoprobes. At MgSO_4_ concentrations of 40 mM and below, the particles treated with compDUS remained stable, as demonstrated by the red-coloured suspensions (**Figure 2A**) and constant SPR at 520 nm (red curves in **Figure 2C**). In contrast, when treated with a non-complementary control oligonucleotide (SDU) the particles were only stable up to 20 mM but aggregated at ≥ 40 mM MgSO_4_, as shown by a colour change from red to blue for all SDU-treated suspensions (**Figure 2B**). At 60 mM MgSO_4_ and above, all nanoconjugate samples aggregated irrespective of the presence of target DNA as shown by the blue suspensions in **Figure 2A**. In agreement with the observed colour changes, the gold nanoparticle SPR red-shifted from 520 nm to longer wavelengths between 143-555 nm (blue curves in **Figure 2C**). This result indicates that the additional stability conferred by target hybridisation was insufficient to withstand the charge screening introduced at higher Mg^2+^ concentrations. It was therefore determined that 40 mM MgSO_4_ was the optimum concentration for identifying whether the nanoprobes have been treated with target DNA and was used henceforth.

### Testing the Sequence Specificity of Aggregation Tests

**Figure 3:**
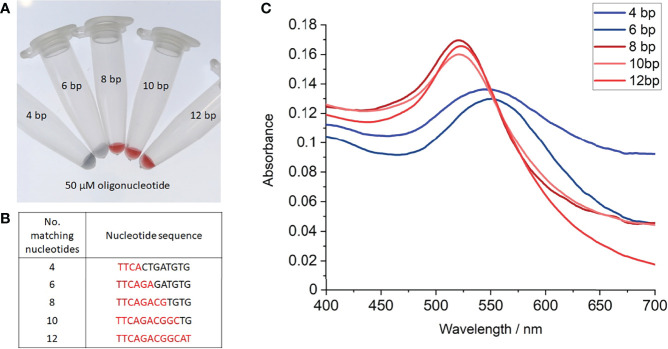
Salt-induced aggregation tests with partial complementary oligonucleotides. **(A)** Photograph of gold nanoprobes after incubation with 50 µM of each oligonucleotide, heating to 95°C, before cooling to room temperature and treating with 40 mM MgSO_4_, **(B)** Table of partial complementary oligonucleotides used, **(C)** UV-vis absorption spectra of nanoprobe samples with each oligonucleotide. Each experiment was repeated 3 times, with photographs shown from 1 repeat.

Oligonucleotides of ≤ 6 complementary bases (nucleotide sequences shown in **Figure 3B**) provided no additional stabilisation to the nanoprobes as indicated by a red-to-blue colour change (**Figure 3A**). In contrast, nanoprobes treated with oligonucleotides composing of ≥ 8 complementary bases did not undergo any colour change (**Figure 3A**), indicating these oligonucleotides are sufficient to stabilise the nanoprobes against 40 mM MgSO_4_. Supporting the observations, UV-vis spectroscopy revealed a red-shift in the nanoprobe SPR to 545-500 nm, when treated with oligonucleotides of ≤ 6 base matches (**Figure 3C**). Together, these results suggest that between 7-8 complementary bases are required for hybridisation to occur between an oligonucleotide and the nanoprobes at room temperature.

### Salt-Induced Aggregation for the Detection of Gonococcal Genomic DNA

**Figure 4:**
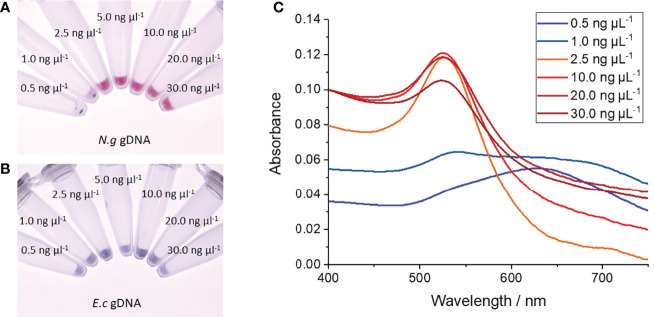
Salt-induced aggregation tests with genomic DNA. **(A)** Photograph of nanoprobes after incubation with increasing concentrations of *N. gonorrhoeae* (MS11) genomic DNA, heating to 95°C, before cooling to room temperature and treated with 40 mM MgSO_4_, **(B)** Photograph of nanoprobes after treatment as before but with *E coli* (Stellar) genomic DNA, **(C)** UV-vis absorption spectra of gold nanoprobe samples following treatment with increasing concentrations of *N. gonorrhoeae* DNA. Each experiment was repeated 3 times, with photographs shown from 1 repeat.

When incubated with *N. gonorrhoeae* (MS11) genomic DNA, the nanoprobes exhibited improved stability at 40 mM MgSO_4_ compared to those incubated with *E. coli (Stellar)* genomic DNA. At gonococcal DNA concentrations of ≥ 2.5 ng µL^-1^, the nanoprobes remained stable at 40 mM MgSO_4_, as demonstrated by their red colour (**Figure 4A**) and constant SPR at 520 nm (red curves in **Figure 4C**). Despite a constant SPR, the absorption intensity of suspension at 30 ng µL^-1^ DNA was unexpectantly lower than observed at the lower concentrations. This was most likely the result of additional charge screening conferred at high DNA concentrations, resulting in low-level particle precipitation. Further studies are required to confirm this; yet regardless of this, no significant plasmon shift or colour change was observed for all samples at ≥ 2.5 ng µL^-1^ *N. gonorrhoeae* DNA, indicating particle stability in the presence of target DNA. In contrast, at DNA concentrations below 2.5 ng µL^-^, visible particle aggregation occurred as indicated by a drastic red-shift in the SPR to 630 nm (blue curves in **Figure 4C**) and a red-to-blue colour change (**Figure 4A**). In addition, some visible particle aggregates began to form 5 minutes after MgSO_4_ addition, as shown by black precipitate in these two suspensions. In contrast, when treated with *E. coli* DNA, the nanoprobes aggregated at all concentrations as shown by distinct blue colour following MgSO_4_ addition (**Figure 4B**). Again, the formation of some visible particle aggregates was also observed, demonstrating high nanoprobe instability in the presence of non-target genomic DNA. Together, these results indicate the DUS-functionalised nanoprobes can specifically detect the presence of gonococcal DNA within solutions with a detection limit of 2.5 ng µL^-1^.

## Discussion

By exploiting the unique optical properties of AuNPs, a multitude of rapid, colour-based diagnostics have been developed for the detection of target DNA sequences (Elghanian et al., 1997; Buckingham et al., 2004; Rosi and Mirkin, 2005; Stoeva et al., 2006; Conde et al., 2010). For this, AuNPs are surface-functionalised with short single-stranded DNA sequences, referred to as oligonucleotides, typically through binding by terminal thiol groups (Rosi and Mirkin, 2005; Carnerero et al., 2017). Due to the highly specific nature of complementary base pairing, the gold nanoprobes can selectively bind to complementary target sequences present in single-stranded DNA or RNA. Hybridisation may either induce particle aggregation or provide additional stability to the particles, depending on the particle DNA-loading (Sato et al., 2003; Song et al., 2010). In this way, aggregation-induced colour changes can be used to indicate the presence of specific target sequences. The first example of this was achieved by Mirkin et al. in 1997 and since then, DNA-functionalised gold nanoprobes have proven useful tools in the facile and rapid detection of pathogenic DNA for the diagnosis of infectious diseases (Elghanian et al., 1997; Liandris et al., 2009; Bakthavathsalam et al., 2012). For this, gold nanoprobes are designed to anneal to a specific sequence found within the target pathogen’s DNA. Such nanoprobes have been successfully employed to detect a range bacterial DNA, including those belonging to *Escherichia coli*, *Mycobacterium tuberculosis* and *N. gonorrhoea*e with typical sensitivities of between 10-250 ng of DNA (Liandris et al., 2009; Bakthavathsalam et al., 2012; Tunakhun et al., 2019). In most examples, the diagnostic requires purified DNA suspensions, and so clinical samples such as a swab or urine require prior sample DNA extraction and purification. Although recently, Alnasser et al. successfully detected the presence of a protozoal parasite directly from urine samples following just centrifugation, filtering and pH adjustment (Alnasser et al., 2016).

In this present study, gold nanoprobes were designed the target the gonococcal DNA Uptake Sequence (DUS), a 12-base sequence present up to 2000 times in *N. gonorrhoeae* genomic DNA (Duffin and Seifert, 2010; Frye et al., 2013). By doing so, it was hypothesised that high specificity would be achieved because the DUS is only found in high frequency within the genomes of *Neisseria* species (Frye et al., 2013). Furthermore, it was proposed that the high DUS frequency within gonococcal genomes, would facilitate multiple nanoprobe-target annealing events, enhancing the sensitivity compared to previous attempts which have focused on the identification of a single repeated sequence (Tunakhun et al., 2019).

**Figure 5:**
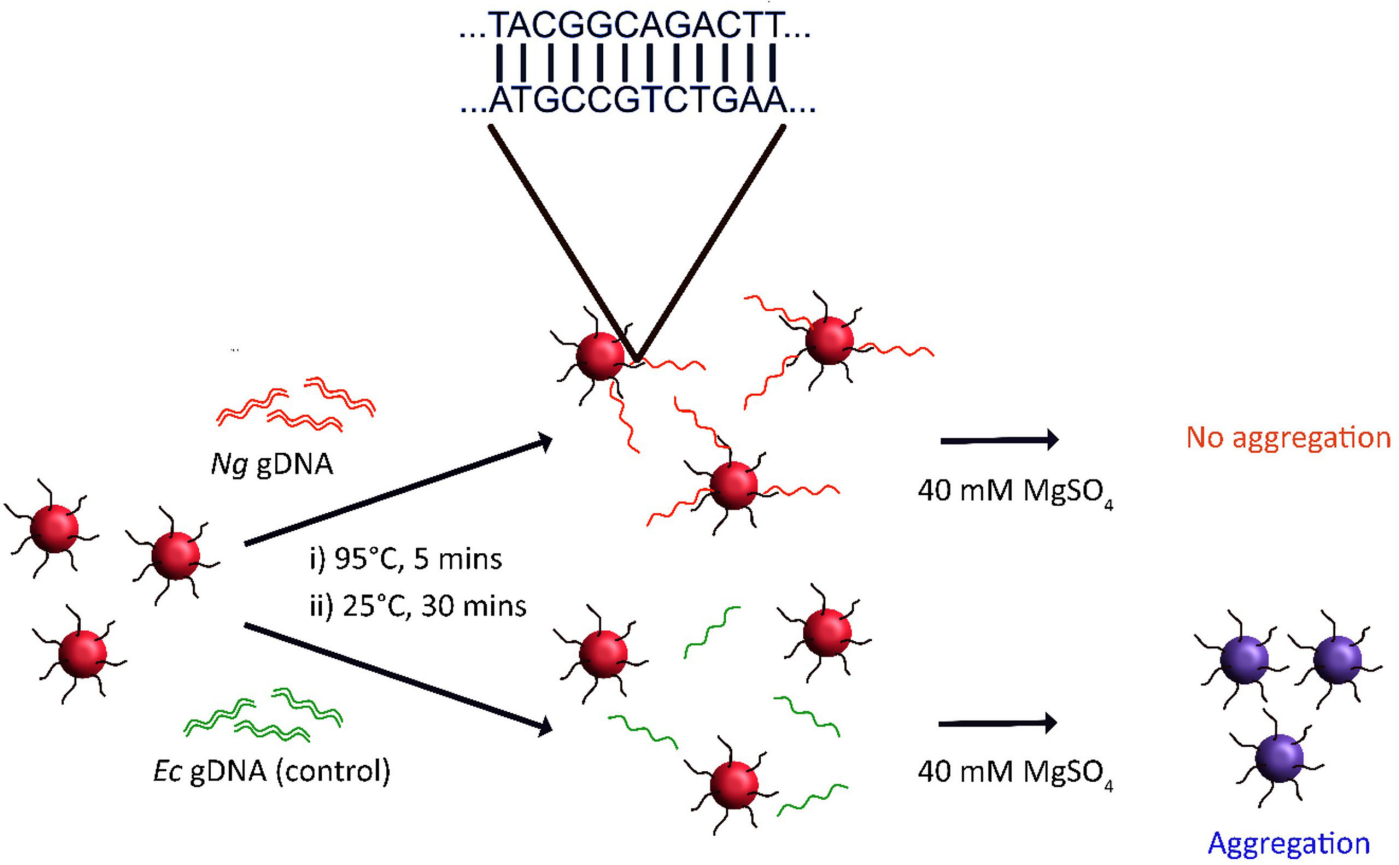
Scheme for *N. gonorrhoeae* (*Ng*) DNA detection *via* salt-induced aggregation of gold nanoprobes. Here, DUS-functionalised nanoprobes are incubated with a test sample of DNA. If the DNA contains the reverse complement DUS then the nanoprobes may anneal to the DNA, producing a double-stranded DNA corona around the nanoprobes. This improves both the steric and electronic stabilisation of the nanoprobes such that they remain stable at higher salt concentrations than nanoprobes which have not been treated with target DNA. *E. coli* (*Ec*) DNA is used as a negative control.

To achieve this, 18.3 nm spherical gold nanoparticles were first prepared, before surface functionalisation with 5’-thiolated oligonucleotides. The single-stranded oligonucleotide contained a single DUS sequence flanked on either side by five random bases. A 10-mer polyA tail was included between the 5’-thiol functionality and base sequence to further enhance the oligonucleotide interaction with the AuNP surface, consistent with observations by Hurst et al. (Hurst et al., 2006). It was proposed that annealing of the nanoprobe to target genomic DNA would confer additional stability to the nanoprobes due to additional steric and electronic stabilization (Song et al., 2010). Hence, it was hypothesised that subsequent salt challenge may be used to identify the presence of target gonococcal DNA (as outlined in the schematic in **Figure 5**), a method previously used to detect other pathogenic bacteria (Baptista et al., 2005; Baptista et al., 2006; Tunakhun et al., 2019).

Our results show that the salt stability of the nanoprobes is improved upon annealing to a reverse complementary oligonucleotide (compDUS). It was found at 40 mM MgSO_4_, nanoprobes that has been treated with compDUS remained stable in solution, whilst those without aggregated leading to a distinct colour change from red to blue. Song et al. previously reported similar stability improvements upon the annealing of nanoprobes with a DNA-loading density below 26 pmol cm^-2^ (Song et al., 2010). Since the nanoprobes used herein have a density of approximately 23.2 pmol cm^-2^ (145 oligonucleotides/AuNP), below this defined threshold, these observations are consistent with Song et al.’s prediction (Song et al., 2010). Testing the oligonucleotide sequence specificity, it was found that only oligonucleotides containing a complementary sequence ≥ 8 bases long were sufficient to stabilise the nanoprobes and provide a positive read-out. Thus, in the context of DNA diagnostics, this result suggests that any DNA which contains sufficient partial DUS matches exceeding 8 bases will likely produce a false-positive result.

Applying the same approach to an extracted and purified suspension of gonococcal DNA, the prepared nanoprobes were shown to successfully detect the presence of gonococcal DNA *via* their hybridisation at multiple complementary DUS sites. At gonococcal gDNA concentrations of ≥ 2.5 ng µL^-1^ the nanoprobe suspension remained red following 40 mM MgSO_4_ challenge. Below this, a red-to-blue colour change was still observed, indicating a detection limit of 2.5 ng µL^-1^, corresponding to 15.0 ng of DNA (1.1 x 10^17^ mol) in the 6 µL of sample. This value is consistent with literature DNA-based diagnostics that have adopted a similar approach. For example, Liandris et al. and Andreadou et al. obtained detection limits of 37.5 ng Mycobacterium DNA and 115.0 ng Leishmania DNA, respectively (Liandris et al., 2009; Andreadou et al., 2014). Furthermore, this result is an 8-fold improvement in sensitivity compared to the closest literature example testing gonococcal DNA, which achieved a detection limit of 20 ng µL^-1^ (120 ng) of DNA by probing for the gonococcal single-copy porB gene (Lynn et al., 2005; Tunakhun et al., 2019). This distinct improvement in sensitivity clearly demonstrates the advantage of targeting a high-frequency DNA sequence such as the DUS (1521 DUS in MS11 *N. gonorrhoeae* genome) rather than a single-copy gene. The detection limit of 15.0 ng of gDNA is equivalent to ∼6.2 million gonococcal cells, which is of a similar order of magnitude to the average bacterial load present in patient urethal swabs (Priest et al., 2017). Therefore, the sensitivity of this diagnostic may be clinically useful, potentially omitting the need for highly demanding and timely cell culture or DNA-amplification methods, such as required for NAATs. Since bacterial loads in alternative patient swabs or asymptomatic patients can be 10-fold lower, further research efforts would be required to further improve sensitivity before clinical use (Chow et al., 2016; Priest et al., 2017; van der Veer et al., 2020). In its current iteration, the diagnostic platform has only been performed on purified DNA samples extracted directly from the test species. Thus, to apply this technique to a clinical test sample such as a swab or urine, the DNA would have to be first extracted and purified from the sample, thereby limiting the technique. Before this detection platform can be extrapolated to clinical settings, direct application of the diagnostic to clinical samples should be tested, or alternatively, a quicker and more affordable DNA-extraction method, compared to the Qiagen DNeasy Blood and Tissue kit may be explored. Despite these limitations, the potential of DUS-targeting gold nanoprobes in *Neisseria* detection has been demonstrated. Crucially, a positive result can be obtained within 30 minutes of application to a DNA sample, compared to NAATs which take between 1-3 days for results to be returned to the patient. Moreover, the visual nature of the readout makes it easy-to-use, negating the need for training or expensive equipment. Hence, this novel approach may offer a distinct speed and affordability advantage over NAATs for use in resource-poor clinics and point-of-care applications.

To assess the specificity for *Neisseria*, the nanoprobe diagnostic was also tested with genomic DNA extracted from an off-target control organism, Stellar *Escherichia coli* (*E. coli, Ec*). *E. coli* was chosen since it is the predominant species found in the uretha, vagina and semen, all of which are common sites of gonococcal infection and thus, are commonly found in patient samples (Solomon and Henkel, 2017).

At all *E. coli* DNA concentrations tested, nanoprobe aggregation occurred. A CBI nucleotide BLAST of the DUS sequence against the Stellar *E. coli* genome, revealed a single complete DUS match with further partial matches (< 12 bp). Since no particle stabilisation was observed using *E. coli* DNA, this finding demonstrates that the relatively low frequency of complete and partial DUS matches within other genomes, compared to the 1521 complete matches in gonococcal DNA, is insufficient to cause a false-positive result. However, since the DUS (or 10-base analogue) is present in high-frequency in all *Neisseria* genomes, any species within the genus could likely provide a positive read-out (Treangen et al., 2008; Frye et al., 2013). Thus, DNA belonging to off-target commensal *Neisseria* present during a a suspected gonococcal infection may lead to a false-positive result. Despite this, the technique still offers a vast improvement from current gram-stain techniques used in resource-poor settings, which can result in false-positives under the presence of any gram-negative diplococci. To further test the specificity of this diagnostic, future validation should be extended both to genomic DNA taken from other members of the *Neisseriaceae* family as well from a range of pathogenic and commensal species frequently found in patient test samples, including urethral or cervical swabs and urine (Ng and Martin, 2005). It may be that the presence of pathogenic *Neisseria* during symptomatic infection, significantly outweigh the presence of commensal flora. This may differ person to person and also by anatomical site of infection. Further studies on clinical specimens correlating our gold nanoprobe platform with species specific PCR will help establish any limitations of this approach to clinical use.

## Conclusions

A gold nanoprobe platform was developed for the colorimetric detection of extracted and purified genomic DNA belonging to *Neisseria* species. The detection protocol is easy-to-use and provides a result in under 30 minutes, potentially making it suitable for the diagnosis of gonococcal infections in resource-poor settings. A detection limit of 15.0 ng was obtained, which corresponds to a clinically relevant gonococcal load. Application of the diagnostic to DNA extracted from an off-target organism provided no read-out, suggesting sensitivity for *Neisseria* species which contain a high frequency of DUS motifs. Together, these results highlight the potential of DUS-targeting gold nanoprobes in the point-of-care diagnosis of gonococcal infections. In particular, these results demonstrate that targeting the high frequency gonococcal DUS motif is a promising approach for enhanced diagnostic sensitivity. In future, the specificity of the diagnostic platform should be further validated by testing against a wide range of off-target species. Moreover, before application as a point-of-care diagnostic, the platform must be extended from operating solely on extracted and purified DNA, into a diagnostic which can be applied directly to patient test samples, or with minimal sample preparation.

## Data Availability Statement

The original contributions presented in the study are included in the article/supplementary material. Further inquiries can be directed to the corresponding authors.

## Author Contributions

EC, DH, and SD contributed to conception and design of this work. EC performed all experiments and wrote the first draft of the manuscript. All authors contributed to manuscript revision, read, and approved the submitted version.

## Funding

PhD studentship funded through the Bristol Centre for Functional Nanomaterials (EPSRC, EP/L016648/1)

## Conflict of Interest

*The authors declare that the research was conducted in the absence of any commercial or financial relationships that could be construed as a potential conflict of interest*.

## Publisher’s Note

All claims expressed in this article are solely those of the authors and do not necessarily represent those of their affiliated organizations, or those of the publisher, the editors and the reviewers. Any product that may be evaluated in this article, or claim that may be made by its manufacturer, is not guaranteed or endorsed by the publisher.
